# 2′-Hydroxychalcone Induced Cytotoxicity *via* Oxidative Stress in the Lipid-Loaded Hepg2 Cells

**DOI:** 10.3389/fphar.2019.01390

**Published:** 2019-11-20

**Authors:** Yun Qian, Yang Yang, Kai Wang, Wenjun Zhou, Yanqi Dang, Mingzhe Zhu, Fenghua Li, Guang Ji

**Affiliations:** ^1^Institute of Digestive Diseases, Longhua Hospital, China-Canada Center of Research for Digestive Diseases (ccCRDD), Shanghai University of Traditional Chinese Medicine, Shanghai, China; ^2^Experiment Center for Science and Technology, Shanghai University of Traditional Chinese Medicine, Shanghai, China; ^3^School of Public Health, Shanghai University of Traditional Chinese Medicine, Shanghai, China

**Keywords:** licorice, 2′-hydroxychalcone, cytotoxicity, reactive oxygen species, hepatocyte

## Abstract

Licorice is a common herb used in traditional Chinese medicine, and has been widely used clinically. Physiologically, although it is relatively safe, licorice-induced hepatotoxicity in the presence of other diseases needs to be evaluated. The present study was conducted to investigate the toxicological effects of the bioactive components of licorice in HepG2 cells cultured with or without free fatty acid (FFA). The compounds, isoliquiritigenin, licorice chalcone A, bavachalcone, and 2′-hydroxy chalcone (2′-HC) inhibited cell proliferation at certain concentrations in lipid loaded cells with limited effects on the normal cells. The representative compound 2′-HC (at a concentration of ≥ 20µM) increased the oxygen consumption rate, ATP production, mitochondrial membrane potential, generation of total and mitochondrial reactive oxygen species (ROS) production, and expression of inflammatory cytokines (TNF-α, IL-6, and IL-8) and Caspase-9 protein; and reduced the expression of SOD1. In addition, we found exaggerated lipid accumulation in HepG2 cells treated with FFA. Our results suggest that 2′-HC at a concentration of ≥ 20µM might cause damage to the hepatocytes. The toxicity may be related to excess ROS production and inadequate SOD1 expression, leading to apoptosis, inflammation, and cellular dysfunctions.

## Introduction

Licorice or liquorice is the root of *Glycyrrhiza glabra* L., which is an herbaceous perennial legume. In some European and Middle-east countries, licorice flavor is used as a sweeteners and food additive, and has been approved by the United States Food and Drug Administration as a food supplement (Michielsen et al., 2018; Alagawany et al., 2019). In China, after extraction and processing, licorice is used in herbalism. It is one of the oldest medicinal herbs, and has been included in the pharmacopoeia of the Asian and European countries (Substances et al., 1974). As a widely used herb, licorice is considered to have the effect of reconciling the medicinal properties of other herbs, and is often included as an auxiliary drug in clinical practice (Li et al., 2019).

In the past few decades, pharmacological studies have suggested that licorice and its bioactive components have the therapeutic effects against inflammation, ulcer, chronic viral infection, hyperlipidemia, and immune dysregulation (Yang et al., 2015). In addition, its major constituent glycyrrhizic acid has been widely used for the treatment of various liver diseases, including hepatitis B, chronic hepatitis, cholestasis, liver fibrosis, liver cancer, etc. (Li et al., 2014). An animal study has shown that glycyrrhizin has a significant protective effects against the development of nonalcoholic steatohepatitis in mice treated with methionine and choline deficiency diet (Yan et al., 2018). Glycyrrhizin can also ameliorate acute liver injury induced by acetaminophen. Besides, it is also suggested that glycyrrhetinic acid has hepatoprotective effects (Jeong et al., 2002; Ji et al., 2016) and has been used for the treatment of liver fibrosis (Moro et al., 2008).

The liver is the main metabolic organ and is also the main site of drug metabolism. It is suggested that stressed hepatocytes are more sensitive to the toxic side effects of drugs (Almazroo et al., 2017). Nonalcoholic fatty liver disease (NAFLD) has become a common disease due to the increased global prevalence of obesity. The typical feature of NAFLD is the accumulated lipids in hepatocytes. Licorice is a frequently used agent as a component of formula such as Sinisan and Ling-gui-zhu-gan decoctionon in preventing and treating NAFLD (Cheng et al., 2017; Dang et al., 2019). While the lipid-loaded hepatocytes are susceptible to chemical toxicity, their resistance to drug toxicity is still unknown.

The main bioactive components of licorice include triterpenoids (glycyrrhizic acid, glycyrrhetinic acid, etc.), flavonoids (isoglycin, isoliquiritn, etc.), alkaloids, polysaccharides, and free phenols (Yang et al., 2015; Li et al., 2019). More than 300 flavonoids have been isolated from licorice including dihydroflavones, dihydroflavonols, chalcone, isoflavones, isoflavones, flavonoids, flavonols, isoflavones, and isoflavones with hydroflavonoids and chalcones as the main types. In the present study, we evaluated the effects of the representative chalcones of licorice on HepG2 cells cultured with or without free fatty acid (FFA), and explored the potential mechanisms of a licorice monomer compound (2′-HC) in the presence of cellular damage.

## Materials and Methods

### Reagents

**Figure 1:**
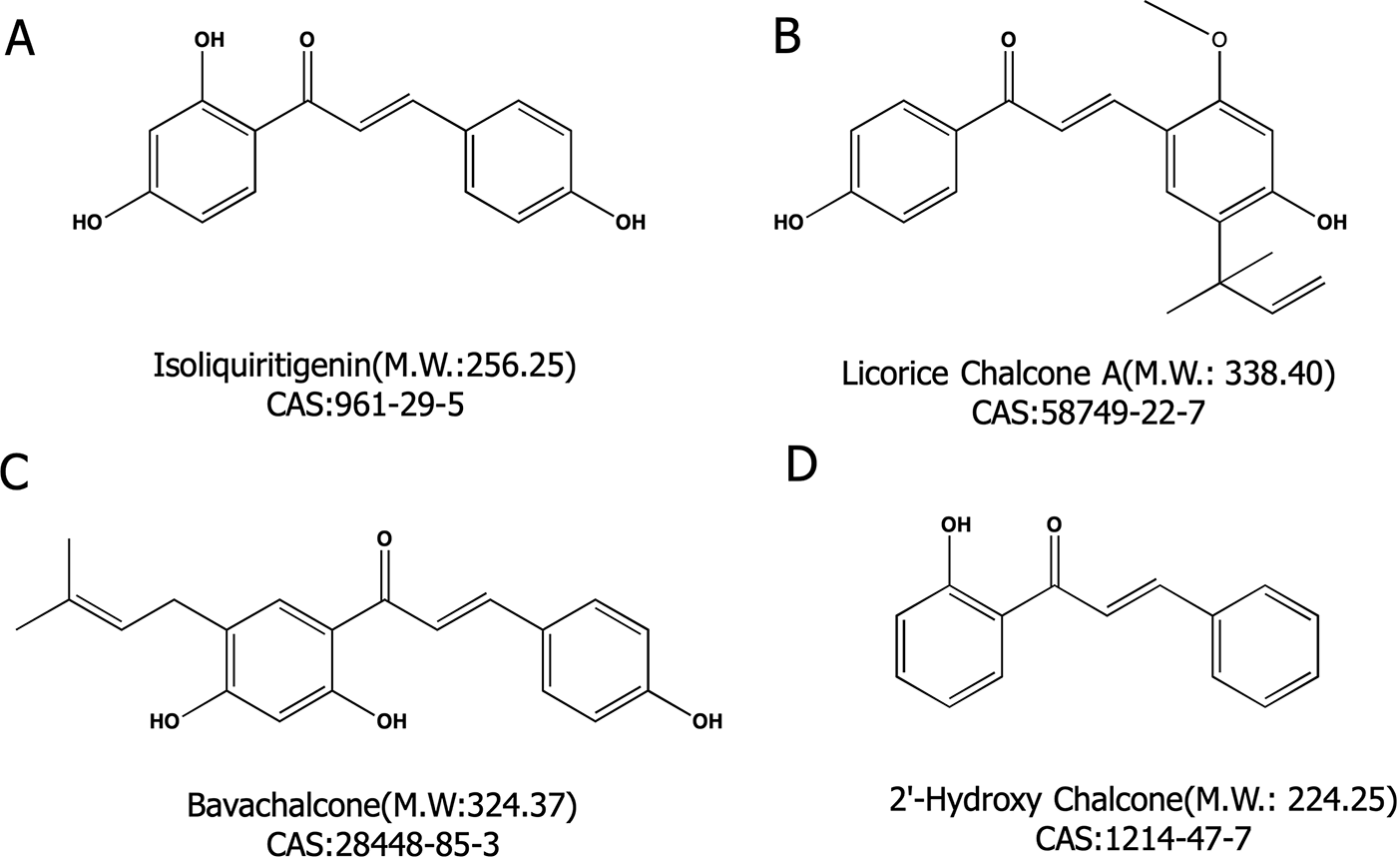
Four chalcone compounds. **(A)** Isoliquiritigenin (2′,4′,4-trihydroxychalcone; ISL) is a flavonoid found in licorice. **(B)** Licochalcone A (LCA) is a characteristic chalcone that is found in licorice. **(C)** Bavachalcone (BC) is a substance with a 2′-HC core structure. **(D)** 2′-HC is the monomeric compound discussed in this paper.

2′-hydroxychalcone (2′-HC, purity > 95%) (**Figure 1D**) and bavachalcone (BC, purity > 98%) (**Figure 1C**) were purchased from Shanghai Yuanye Biological Technology Co, Ltd. Isoliquiritigenin (LSL, purity ≥ 98%) (**Figure 1A**) and licorice chalcone A (LCA, purity ≥ 98%) (**Figure 1B**) were purchased from Dalian Meilun Biological Technology Co, Ltd. Oleic acid (OA) and Palmitic acid (PA) were purchased from Sinopharm Chemical Reagent Co, Ltd. (Shanghai, China).

### Cell Culture and Treatment

The HepG2 human hepatocytes were grown in Dulbecco’s modified Eagle’s medium (DMEM) containing 10% fetal bovine serum (FBS, Gibco) and 1% antibiotic (100 U/ml penicillin and 100 ug/ml streptomycin, Gibco, USA) at 37°C in a humid atmosphere of 5% CO_2_. The cells were harvested and seeded at an initial density of 1 × 10^5^ cells/ml onto the bottom of 96-well plates for experimental use. The cell density of a 6-well culture plate was 1.5 × 10^5^ cells/ml. A special 96-well culture plate (Seahorse Bioscience) with a cell density of 8 × 10^4^ cells/mL was used to detect the cellular respiratory function. A combination of OA and PA (2:1) was dissolved in DMEM medium (Gibco, USA) to induce NAFLD in HepG2 cells.

### MTT [3-(4,5-Dimethylthiazol-2-Yl)-2,5-Diphenyltetrazolium Bromide] (MTT) Assay

MTT (Sangon Biotech, Shanghai, China; 20 µL; 0.25 mg/ml) was added to each well and the cells were incubated for 4 h. The supernatants were removed and 100 µL DMSO was added to each well, and the plates were shaken for 5 min. The absorbance was measured at 570 nm by a microplate reader (Molecular Devices, USA).

### Crystal Violet Assay

After removing the supernatants, the cells were washed twice and stained by 50 µL/well crystal violet solution for 15 min. Each well was rinsed with running water and dried. Crystal violet (100 µL) solution was added to each well, and the plates were shaken for 5 min. The absorbance was measured at 540 nm and 630 nm by a microplate reader (molecular Devices, USA).

### Microplate Detection Analysis

The HepG2 cells in the logarithmic growth phase were adjusted to 1.5×10^5^ cells/ml and inoculated in 6-well plates. After adhering for 24 h, 0.3 mM free fatty acid was added for modeling, and the same amount was added to the control group. DMEM was added to 2′-HC at a final concentration of 10, 20, and 40 µM, and the supernatant and the cells were collected after 24 h of culture. After the treatments, the triglyceride (TG) content, lactate dehydrogenase (LDH) activity, adenine nucleotide triphosphate (ATP) content, malondialdehyde (MDA) activity and nicotinamide adenine dinucleotide (NADH) activity were estimated according to the kit instructions (Jiancheng, Nanjing, China).

### Estimation of Lipid Accumulation Within Cells

Oil red O staining is commonly used to stain lipids and demonstrate hepatic steatosis. The cells were cultured with FFA along with various concentrations of 2′-HC overnight. The cells were then fixed with 10% neutral formaldehyde for 1 h, and stained with oil red O for 20 min followed by hematoxylin for 3 min. After washing with PBS, the cellular morphology and lipid accumulation were observed under the microscope (Advanced Microscopy Group, USA).

### Detection of Intracellular ROS Detection

Dichlorodihydrofluorescein diacetate(DCFH-DA) probe and MitoSOX™ Red Mitochondrial Superoxide Indicator were used to detect the total and mitochondria-derived ROS. The cells were seeded in 6-well plates with various concentrations of 2′-HC and FFA and washed once with PBS. The cells were incubated with either DCFH-DA probe (10 µM, Dalian Meilun Biological Technology Co, Ltd. China) or MitoSOX™ reagent (5 µM, Thermo Fisher Scientific, USA) and Hoechst 33342 (0.5 µg/mL, Invitrogen, USA) at 37°C for 30 min. After the staining, the excess dye was washed away and the analysis was carried out using Image Xpress Micro 4 (Molecular Devices, USA).

### Flow Cytometry Analysis

Apoptosis and mitochondrial membrane potential changes were analyzed by flow cytometry. The cells were collected by trypsinization (250 g, 4°C, and 5 min) and washed twice with cold PBS. Apoptosis analysis was performed using the BD FACSCalibur™ flow cytometer (BD Biosciences, USA) according to the instructions of FITC Annexin V Apoptosis Detection Kit I (BD Biosciences, USA). The experimental procedure for the detection of membrane potential was the same as that of detecting apoptosis and the cells were stained with JC-10 (Dalian Meilun Biological Technology Co, Ltd. China). The results were analyzed by FlowJo software.

### Cellular Oxygen Consumption Rate (OCR) and Extracellular Acidification Rate (ECAR) Assay

The cellular respiratory function promoted by 2′-HC was measured using an XF96 Extracellular Flux analyzer (Seahorse Bioscience, North Billerica, MA). After the cells were treated with FFA (0.3 mM) and 2′-HC with different concentrations overnight, the medium was discarded, replaced with a dedicated medium for detection, and incubated for 1 h at 37°C in a carbon dioxide-free environment. The OCR and ECAR assays were performed in real-time by injecting oligomycin (0.5 µM), carbonyl cyanide-4 (trifluoromethoxy) phenylhydrazone (FCCP, 0.5 µM), and rotenone + antimycin A (0.5 µM) at indicated time points according to the instructions of XF Cell Mito Stress Test Kit (Seahorse Bioscience, USA).

### Real-Time Quantitative Reverse Transcription Polymerase Chain Reaction (Rt-Pcr)

Real-time quantitative RT-PCR was used to determine the relative expression of mRNAs. According to the manufacturer’s instruction, the total RNA was isolated from the cells using RNAiso Plus (Takara, Japan), and cDNA was synthesized using PrimeScript™ RT Master Mix (Takara, Japan) by Biometra Tone Cycler (Analytixjena, German). Real-time quantitative PCR was performed using TB Green™ *Premix Ex Taq*™ (Takara, Japan) and the 7500 fast Real-Time PCR Systems (Applied Biosystems, USA). The primer sequences were obtained from Invitrogen (Shanghai, China) and listed in **Table 1**. The gene expression was normalized by β-actin.

**Table 1:** Primer sequence.

Primer name	Primer sequence (5′-3′)
	F: CATGTACGTTGCTATCCAGGC
β-actin	
	R: CTCCTTAATGTCACGCACGAT
CYCs	
	R: TTATTGGCGGCTGTGTAAGAG
IL-8	
	R: AACCCTCTGCACCCAGTTTTC
IL-6	
	R: CCATCTTTGGAAGGTTCAGGTTG
TNF-α	
	R: CGGGCCGATTGATCTCAGC
IL-lβ	
	R: TTTTTGCTGTGAGTCCCGGAG
BAX	
	R: CCAGCCCATGATGGTTCTGAT
BCL-2	
	R: CGGTTCAGGTACTCAGTCATCC
Caspase-3	
	R: CTACAACGATCCCCTCTGAAAAA
Caspase-9	
	R: GCATTTCCCCTCAAACTCTCAA
Caspase-7	
	R: CGGCATTTGTATGGTCCTCTT
Caspase-6	
	R: AGGAGGAGCCATATTTTCCCA

### Western Blotting Analysis

The hepatocytes were lysed with ice-cold RIPA buffer and the protein extraction was immunoblotted to analyze the protein expression. The protein lysates were separated by SDS-PAGE and transferred onto polyvinylidene fluoride membranes (Merck Millipore Co, Ltd. USA). Immunoblotting was performed according to the standard procedures with the following primary antibodies: β-actin (4,970, 1:1,000), BAX (5,023, 1:1,000), BCL-2 (2,870, 1:1,000), Caspase-3 (9,665,1:1,000), SOD2 (13,141,1:1,000), and Cytochrome C (11,940, 1:1,000) antibodies [Cell Signaling Technology (Beverly, MA, USA)], antibodies for SOD1 (ab13,498,1:5,000) and Caspase-9 (ab219590, 1:1,000) [obtained from Abcam (Cambridge, MA, USA)], and goat anti-rabbit IgG (H + L) HRP (BS13278, 1:5,000) [obtained from Bioworld Technology (St. Paul, MN, USA)]. The membranes were exposed and visualized using the ECL immobilon western chemiluminescent HRP substrate (WBKLS0500, Millipore, USA). Quantitative analysis was performed using Quantity One software (Bio-Rad Laboratories).

### Statistical Analyses

The results are expressed as the mean ± SD. The data were compared using one-way ANOVA followed by Tukey’s test. GraphPad Prism 6.0 was used for the statistical analyses. A *p*-value of < 0.05 indicated statistical significance.

## Results

### Lipid-Loaded Hepatocytes Were More Sensitive to 2′-HC and Its Derivatives

**Figure 2:**
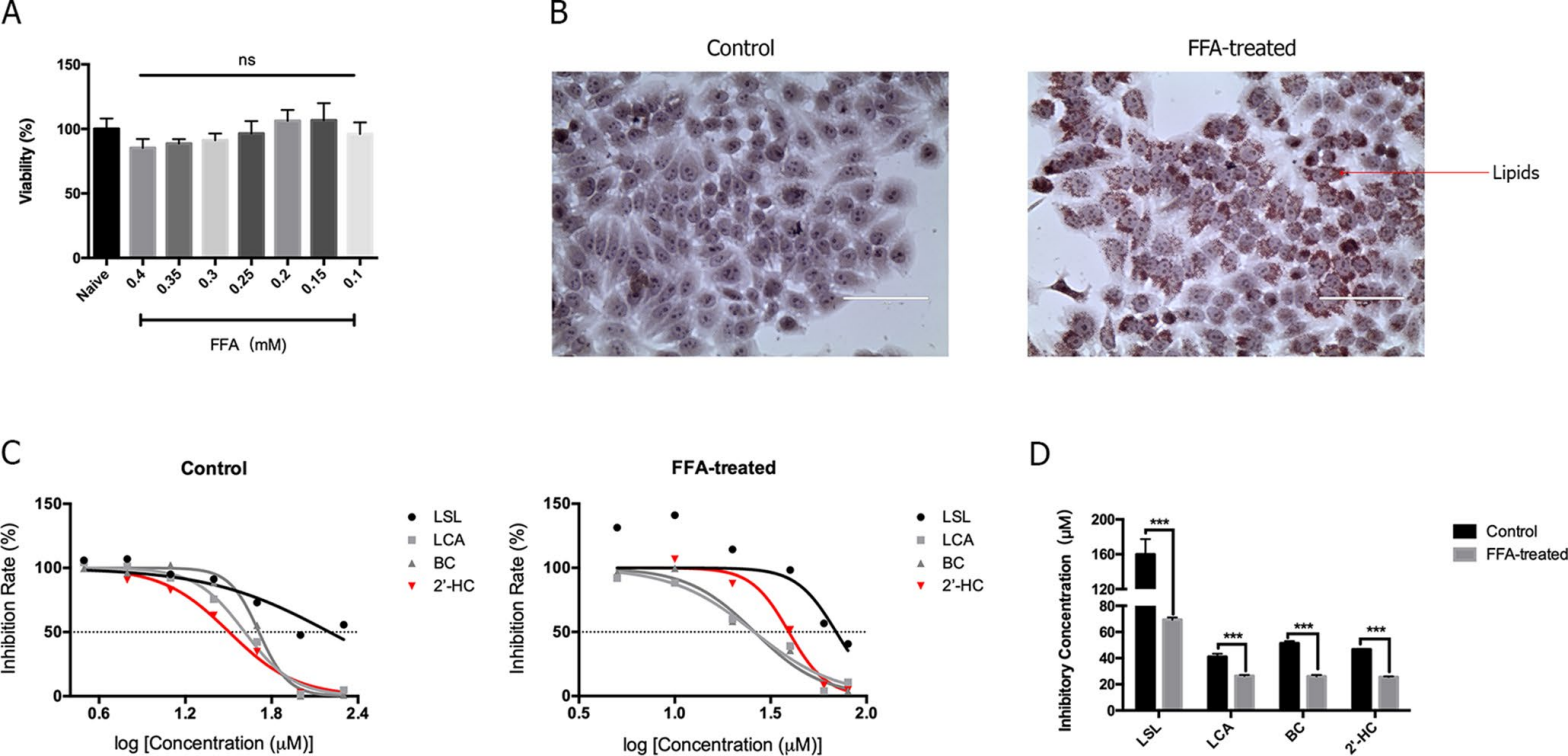
The lipid-loaded hepatocytes were more sensitive following treatment with 2′-HC and its derivatives. **(A)** Effects of different concentrations of FFA on cell viability. **(B)** Effects of FFA (0.3mM) on lipid droplet formation by Oil Red O staining. **(C)** Effects of different chalcone compounds on cell viability. **(D)** IC_50_ of the chalcone compounds. The data are shown as the mean ± SD, ***p < 0.001. ns, no statistical difference.

Hepatic steatosis was characterized by the accumulation of lipids within the hepatocytes and is thought to be in a reversible stage. FFA is commonly used to induce steatosis *in vitro*. As shown in **Figure 2A**, low concentrations (0.1-0.4 mM) of FFA had no significantly effects on cell viability. Morphologically, lipid droplets could be found within the HepG2 cells when cultured with 0.3 mM of FFA (**Figure 2B**). The toxicity of chalcones, including LSL, LCA, BC, and 2′-HC, was evaluated in the HepG2 cells cultured with and without FFA. The chalcones had no or limited effects on the viability of HepG2 cells cultured in the common medium. When the HepG2 cells were treated with FFA, chalcones could reduce the IC_50_ value (**Figure 2C**). Of the four chalcone compounds, the IC_50_ value was significantly reduced in HepG2 cells co-cultured with FFA in comparison to common the medium culture (**Figure 2D**). It is worth noting that 2′-HC is the parental structure and an intermediate metabolite of other chalcone compounds, and hence, we selected 2′-HC as the representative compound for the following experiment.

### 2′-HC Induced Cytotoxicity in the Lipid-Loaded Hepatocytes

**Figure 3:**
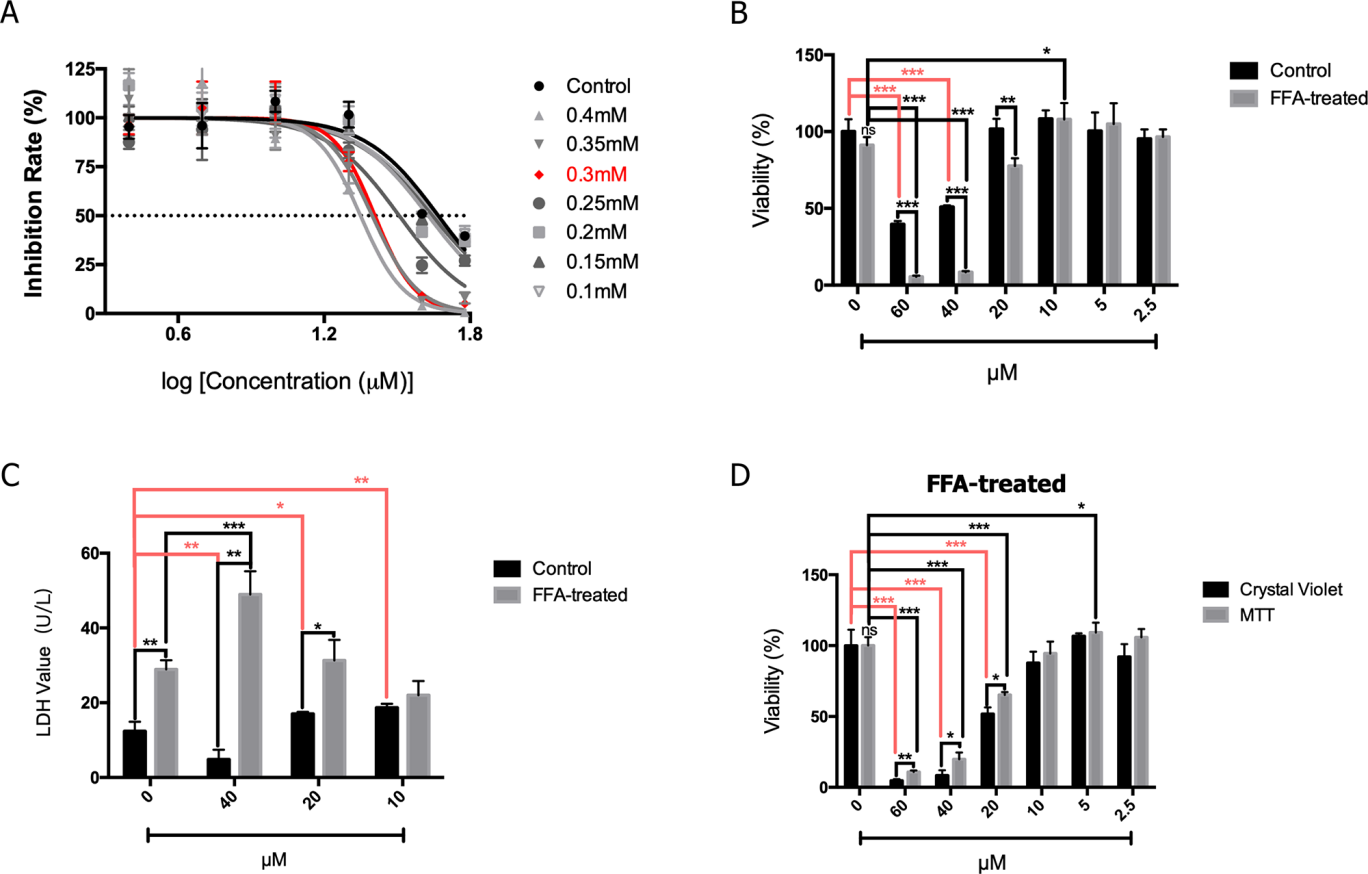
2′-HC exacerbated damage in the lipid-loaded hepatocytes. **(A)** Inhibitory effects of different concentrations of 2′-HC treated with FFA. **(B)** Effects of different concentrations of 2′-HC on cell viability. **(C)** Effects of different concentrations of 2′-HC on the LDH level. **(D)** Different methods for detecting cell viability. The data are shown as the mean ± SD, *p < 0.05, **p < 0.01, ***p < 0.001.

As shown in **Figure 3A**, the HepG2 cells were treated with FFA and the effects of different concentrations of 2′-HC were assessed. The rate of inhibition of 2′-HC was increased with an increased FFA concentration. As previously indicated, 0.3 mM of FFA was a proper concentration to induce lipid accumulation within the HeG2 cells. Under this condition, the cell viability was significantly inhibited with 2′-HC (20 µM, 40 µM, and 60 µM) treatment (**Figure 3B**). The effect was further validated during LDH detection (**Figure 3C**), and both 20 µM and 40 µM 2′-HC increased the LDH level in the lipid-loaded cells, indicating potential cell damage. We compared MTT and crystal violet assays in evaluating cell viability, and confirmed the toxicity of 2′-HC and its concentrations (**Figure 3D**).

### 2′-HC Promoted Cell Respiration in the Lipid-Loaded Hepatocytes

**Figure 4:**
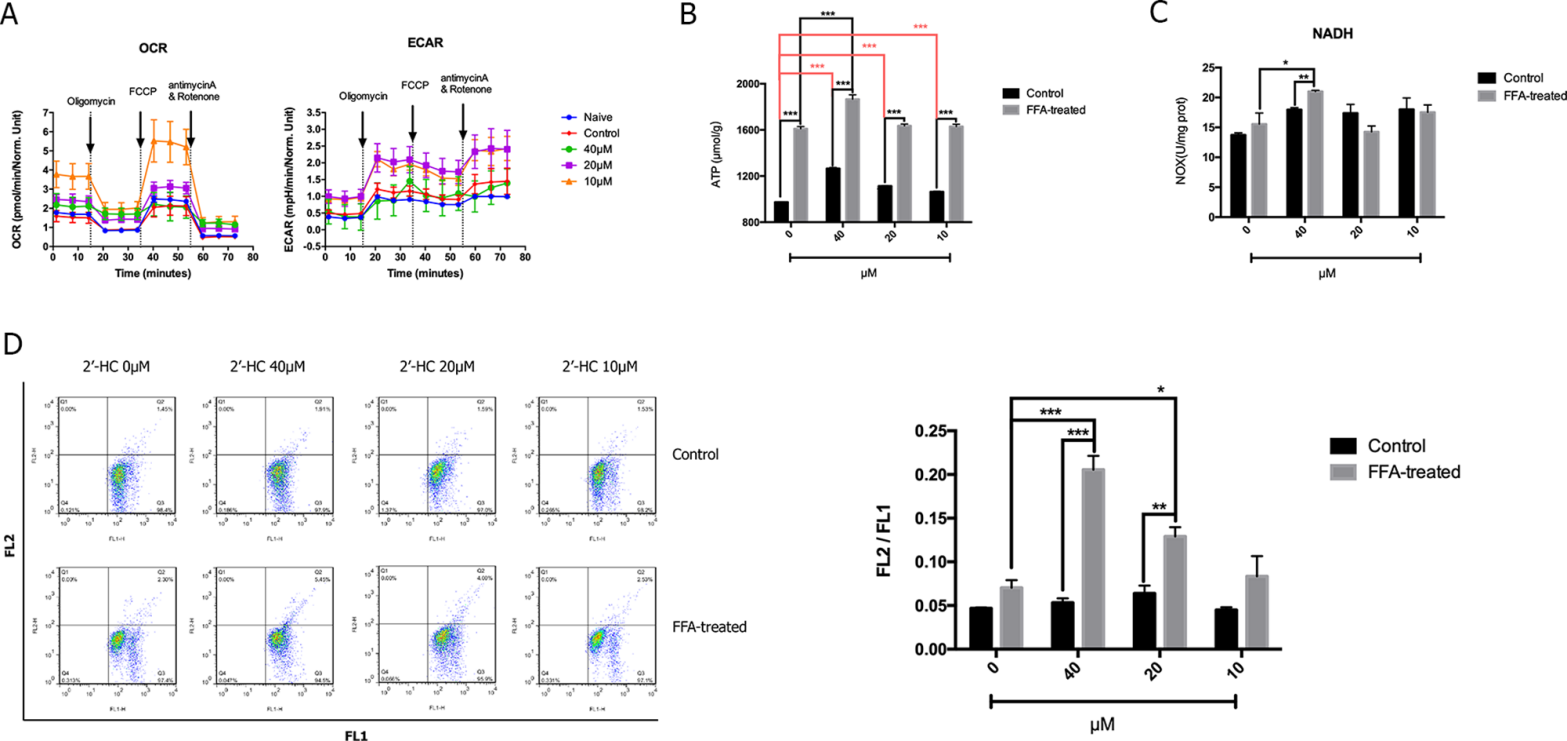
2′-HC promoted cell respiration within the lipid-loaded hepatocytes. **(A)** Effects of different concentrations of 2′-HC on cell oxygen consumption and extracellular acidification rate. **(B)** Effects of different concentrations of 2′-HC on ATP production. **(C)** Effects of different concentrations of 2′-HC on NADH. **(D)** Effects of different concentrations of 2′-HC on mitochondrial membrane potential. The data are shown as the mean ± SD, *p < 0.05, **p < 0.01, ***p < 0.001.

Since cell viability is closely associated with cellular respiratory function, we studied the effect of 2′-HC on the cellular respiratory function. Seahorse XF analyzer was used to detect OCR (an indicator of monitoring oxidative respiration) and ECAR (an indicator of monitoring glycolysis). As shown in **Figure 4A**, the respiratory function of the FFA-treated cells did not change significantly as compared to that of the negative control cells cultured without FFA. 2′-HC increased OCR and ECAR of the cells, indicating that 2′-HC can promote respiratory function in the FFA-treated cells. As a metabolic substrate of the cellular respiratory chain, FFA significantly increased ATP production, which can be further promoted by 2′-HC treatment in the FFA-treated cells (**Figure 4B**). NADH dehydrogenase is closely associated with the mitochondrial function and oxidative stress. However, the NADH value did not change upon 2′-HC treatment (**Figure 4C**). The mitochondrial membrane potential is a prerequisite for the production of ATP, which can adequately reflect the activity of the entire mitochondrial function. Thus, changes in mitochondrial membrane potential in cells were detected, which also suggests the enhancing effects of 2′-HC on the cellular respiratory function (**Figure 4D**). FFA increased the mitochondrial membrane potential in the cells while 2′-HC showed a dose-dependent effects on the FFA-treated cells. These results indicate that 2′-HC may promote cellular respiratory metabolisms, such as oxygen consumption in aerobic respiration and stimulate the release of H^+^ during glycolysis.

### 2′-HC Promoted Oxidative Stress in the Lipid-Loaded Hepatocytes

**Figure 5:**
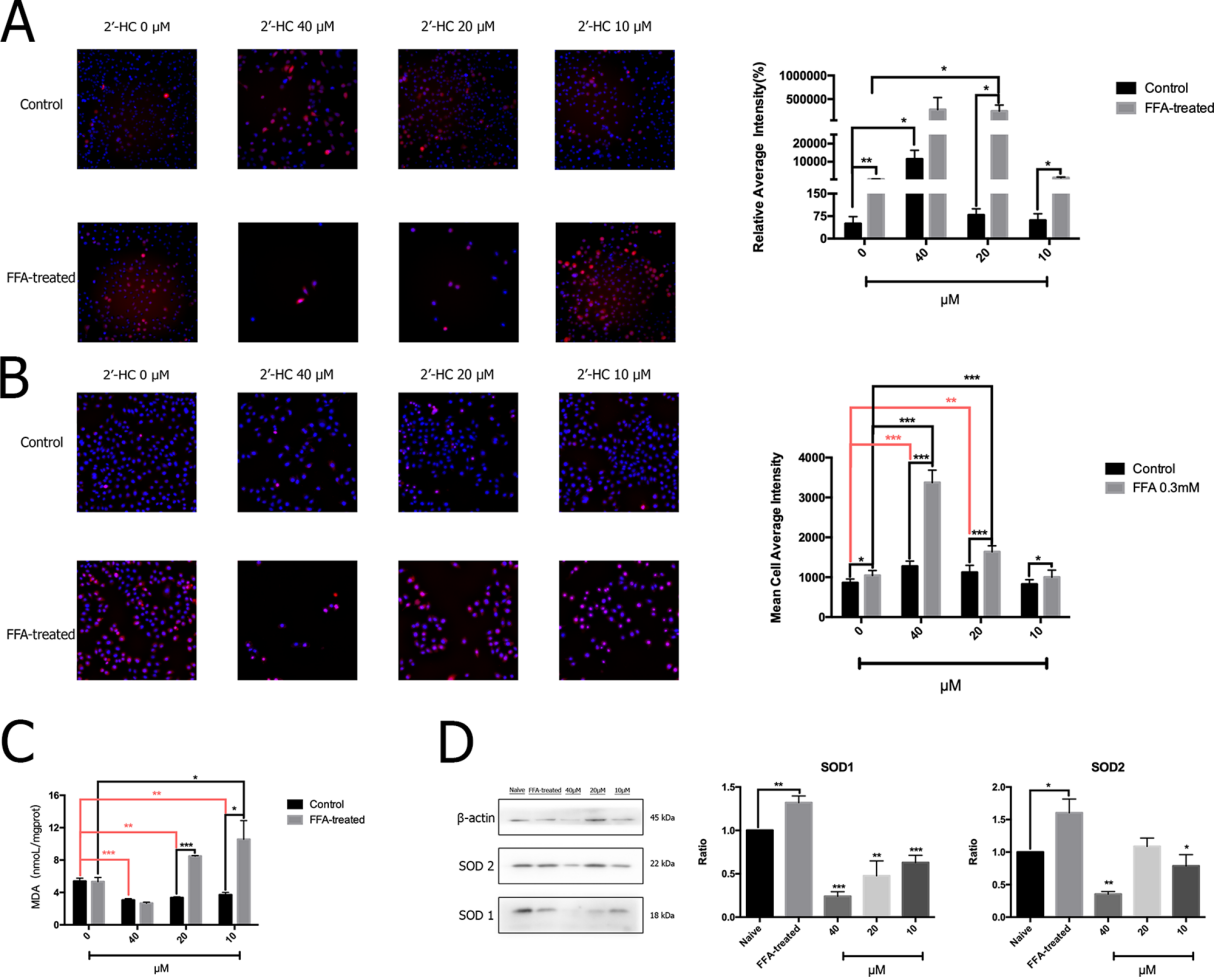
2′-HC promoted oxidative stress in the lipid-loaded hepatocytes. **(A)** Effects of different concentrations of 2′-HC on the total ROS level. **(B)** Effects of different concentrations of 2′-HC on the mitochondrial-derived ROS level. **(C)** Effects of different concentrations of 2′-HC on MDA. **(D)** Effects of different concentrations of 2′-HC on SOD protein expression. The data are shown as the mean ± SD, *p < 0.05, **p < 0.01, ***p < 0.001.

The production of ROS may increase when the cellular respiratory function and energy metabolism increases. The oxidative stress response is further induced when there is an imbalance between the production and elimination of ROS. The total ROS in the cells was detected by specific fluorescent markers, and high-content analysis revealed that 2′-HC (20 µM and 40 µM) significantly increased the relative fluorescence intensity of ROS in the FFA-treated cells (**Figure 5A**). Through MitoSOX™ assay, we also found that 2′-HC dose-dependently increased mitochondrial-specific ROS production (**Figure 5B**). MDA is one of the most important products of membrane lipid peroxidation, and an indicator of cell damage. We noticed that 2′-HC (10 µM and 20 µM) increased the MDA level in the FFA treated hepatocytes (**Figure 5C**). SOD is an important antioxidant enzyme. By detecting the relevant protein expression, we found that 2′-HC reduced the SOD1 and SOD2 expression in the FFA-treated HepG2 cells, especially at a concentration of 40 µM (**Figure 5D**). The above data showed that 2′-HC deranged the oxidant and anti-oxidant balance.

### 2′-HC Caused Apoptosis of the Lipid-Loaded Hepatocytes

**Figure 6:**
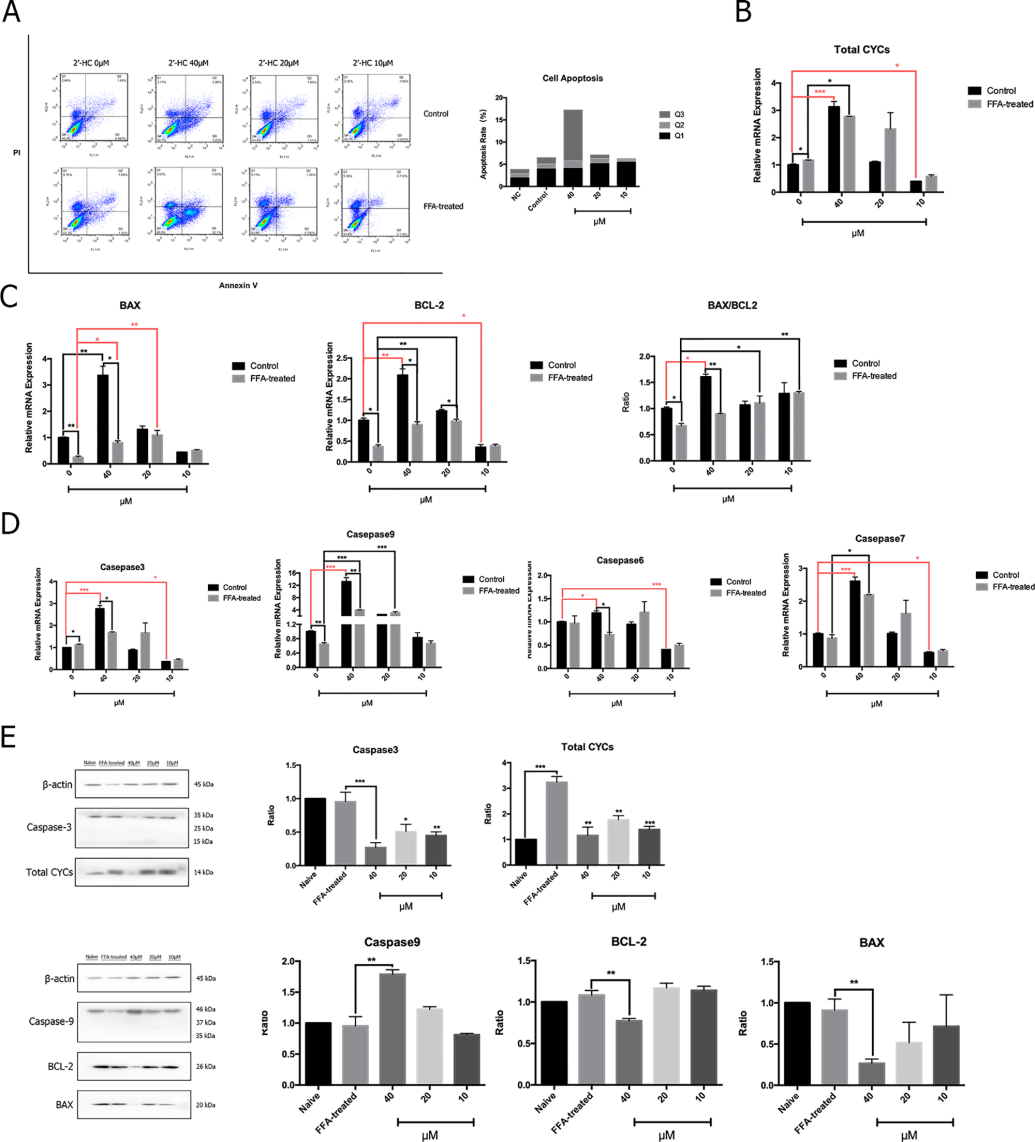
2′-HC caused apoptosis of lipid-loaded hepatocytes. **(A)** Effects of different concentrations of 2′-HC on apoptosis by flow cytometry. **(B)** Effects of different concentrations of 2′-HC on the total Cytochrome C mRNA expression. **(C)** Effects of different concentrations of 2′-HC on BAX and BCL-2 mRNA expression. **(D)** Effects of different concentrations of 2′-HC on mRNA expression of Caspase-3, 6, 7, and 9. **(E)** Effects of different concentrations of 2′-HC on total Cytochrome C, BAX, BCL-2, Caspase-3 and 9 protein expression. The data are shown as the mean ± SD, *p < 0.05, **p < 0.01,***p < 0.001.

Abnormal cell metabolism leads to ROS production and resulting in the changes in apoptosis-related gene targets and activation of apoptosis programs. Therefore, flow cytometry was used to detect the effects of different concentrations of 2′-HC on apoptosis of the FFA-treated cells (**Figure 6A**). The proportion of apoptosis of the FFA-treated cells was higher as compared to the control, and the apoptosis rate was directly proportional to the concentration of 2′-HC. To assess the effect of 2′-HC on apoptotic pathways, several relevant mRNA levels were investigated. Cytochrome C (CYC) is an important carrier of neutrons in the mitochondrial respiratory chain. When CYC is released from the mitochondria into the cytosol, the caspase family and other pathways can be triggered to induce apoptosis. As shown in **Figure 6B**, 20 µM of 2′-HC significantly increased the mRNA expression of the total CYC in the FFA-treated HepG2 cells. 2′-HC significantly increased the mRNA expression of BAX and BCL-2 in the FFA-treated HepG2 cells. In addition, The BAX/BCL-2 expression ratio increased as the drug concentration was reduced (**Figure 6C**). As shown in **Figure 6D**, 20 µM and 40 µM of 2′-HC increased the mRNA expression of Caspase-7 and Caspase-9 in the FFA-treated HepG2 cells. Moreover, the protein expression of Caspase-9 upon 2′-HC treatment was increased (**Figure 6E**), confirming the role of 2′-HC in regulating apoptosis of the lipid-loaded hepatocytes.

### 2′-HC Aggravated Inflammation and Lipid Stress in the Lipid-Loaded Hepatocytes

**Figure 7:**
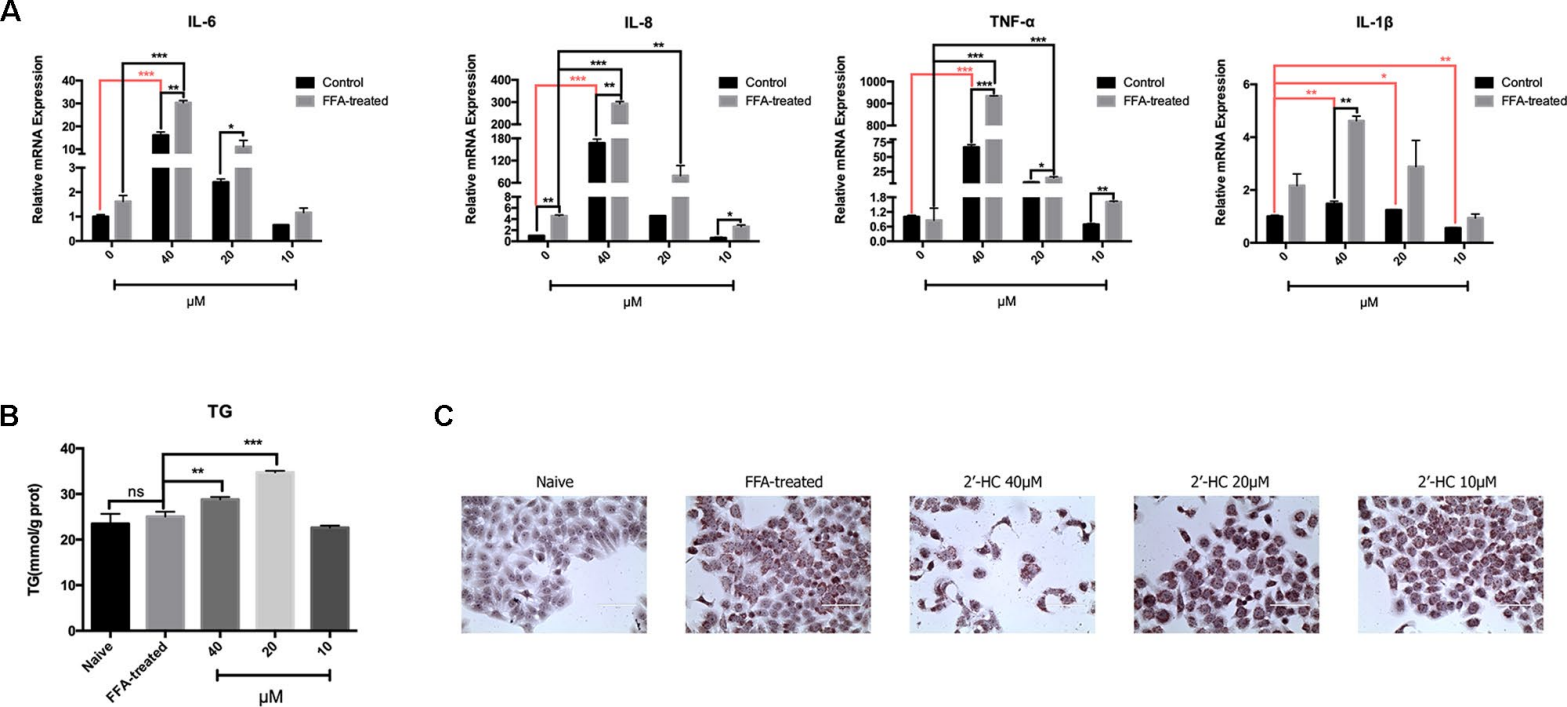
2′-HC aggravated inflammation and lipid stress in the lipid-loaded hepatocytes. **(A)** Effects of different concentrations of 2′-HC on the cytokines. **(B)** Effects of different concentrations of 2′-HC on TG. **(C)** Effects of different concentrations of 2′-HC on lipid droplet formation by Oil Red O staining. The data are shown as the mean ± SD, *p < 0.05, **p < 0.01, ***p < 0.001. ns, no statistical difference.

Oxidative stress is a key inducer of inflammation. Therefore, the expression of the relevant inflammatory cytokines transcription was evaluated. After co-culturing with 2′-HC, the mRNA expression of IL-1β, TNF-α, IL-6, and IL-8 significantly increased in the FFA treated HepG2 cells in comparison to those cultured in the common medium (**Figure 7A**). Besides, 2′-HC promoted lipid accumulation in the FFA-treated hepatocytes. As shown in **Figure 7B**, 2′-HC at concentrations of 20 and 40 µM increased TG in the FFA-treated cells. Oil red O staining also showed an obviously increased in the lipid droplets in the 2′-HC treated cells as compared to the controls (**Figure 7C**).

## Discussion

Drugs are an important cause of hepatotoxicity. A multi-center retrospective survey of the European and Brazilian poison center revealed that licorice was one of the most ten frequently reported plants causing adverse effects (LüDe et al., 2016). In the present study, we found that chalcone, the main ingredient of licorice, was toxic to the lipid-loaded hepatocytes, and the toxicity might be related to the active cell respiration and oxidative stress, leading to cellular apoptosis and inflammation.

We found that chalcone compounds (LSL, LCA, BC, and 2′-HC) in licorice dose-dependently reduced the viability of the cells and increased the level of LDH released from the lipid-loaded hepatocytes, suggesting the hepatoxic potential of the compounds. Studies have demonstrated that the mechanisms of hepatoxicity are various, including the formation of enzyme-drug adducts that may cause immune response and damage to the cells (Beaune et al., 1987; Robin et al., 1997), inhibition of hepatic drug transformation and metabolism (Honig et al., 1992; Yun et al., 1993), prevention of bilirubin secretion, etc. Dysfunctions of the mitochondria and apoptosis are also frequently reported mechanisms responsible for drug-induced hepatoxicity (Pessayre et al., 2001). Here we observed that 2′-HC significantly promoted OCR and EACR in the lipid-loaded hepatocytes, suggesting that the respiration of the cells was active upon 2′-HC treatment. The liver is the main organ metabolizing drugs, and the transformational and metabolic process requires a large amount of energy (Almazroo et al., 2017). Consistent with active respiration, the ATP production was also increased in the lipid-loaded HepG2 cells treated with 2′-HC. However, we are uncertain whether the number of mitochondria plays a role in this process.

ROS are derived from the metabolism of oxygen as by-products of cellular respiration (Imen Belhadj et al., 2014). Physiologically, anti-oxidants, such as SOD could combat the oxidative agent to maintain the dynamic balance. Once the balance is destroyed, excess ROS might cause damage to the cells (Lobo et al., 2010). It is reported that the cytotoxicity of acetaminophen and ethanol was associated with ROS overproduction and mitochondrial Hsp70, which might be mediated through CYP2E1 (Laetitia et al., 2011). Non-steroidal anti-inflammatiory drugs (NSAIDs), especially diclofenac, are often found to induce cytotoxicity to multiple organs. The dysfunction of the mitochondria and the increased production of ROS are the main causes of the hepatoxicity (Han et al., 2019). In our study, both total mitochondrial ROS and MDA were increased in the lipid-loaded HepG2 cells treated with 2′-HC, while the expression of SOD1 was decreased, indicating the imbalance between the two systems along with possible oxidative stress due to 2′-HC treatment.

ROS, especially mitochondrial ROS, is actively involved in regulating immune response and inflammasome activation. It is revealed that mitochondrial ROS can directly activate NACHT, LRR, and PYD domain-containing protein 3 (NLRP3) inflammasome; promote caspase 1 activation and maturation; and subsequently lead to the release of the proinflammatory mediators, such IL-1β (Fumitake et al., 2015). Our results indicate that 2′-HC promoted ROS production and stimulated the release of inflammatory factors, such as TNF-α, IL-6, IL-1β, and IL-8. These data suggest that 2′-HC had toxic effects on the FFA-treated HepG2 cell model which is associated with the inflammation.

Licorice is widely used as the most commonly traditional Chinese medicine, and many health products contain licorice or its ingredients. A report of the European Union indicates a series of symptoms, such as hypertension, muscle weakness, and headache which may appear upon licorice overdose. The herb is also being used in TCM prescriptions for treating various diseases, including NAFLD. Chalcone compound is a major component of the active components of licorice, and has a potential risk of causing cell damage and lipid accumulation within the hepatocytes. Therefore, the licorice should to be cautiously used in the clinics, and the dosage needs to be comprehensively considered. Our data provided a preliminary idea of the safety profile of licorice for the suitable medical use in the future.

In summary, the present study evaluated the toxicological effects of the bioactive components of licorice, and focused on the toxic effects of 2′-HC on the lipid-loaded HepG2 cells. We demonstrated that 2′-HC promoted cellular respiration, ROS production, oxidative stress, inflammation and apoptosis. However, our experiments were limited to *in vitro* cells. The hepatotoxicity of licorice *in vivo* may also be affected by the interactions of various compounds, absorption and metabolism of the compound, tissue distribution, physiological and pathological states, etc. (Luís et al., 2018). Concurrent administration of licorice with NSAIDs, insulin, aspirin, etc. should be paid special attention. Notably, future studies are necessary to further evaluate the adverse effects of licorice, especially in the presence of pathological conditons.

## Data Availability Statement

The raw data supporting the conclusions of this manuscript will be made available by the authors, without undue reservation, to any qualified researcher.

## Author Contributions

FL and GJ designed experiments. YQ, YY, and KW performed the experiment. WZ and YD analyzed data. YQ and MZ drafted the manuscript along with the figures and tables.

## Funding

This work was supported by the National Natural Science Foundation of China (81620108030, 81804018, and 81973554).

## Conflict of Interest

The authors declare that the research was conducted in the absence of any commercial or financial relationships that could be construed as a potential conflict of interest.
